# Impact of centralization of prostate cancer services on the choice of radical treatment

**DOI:** 10.1111/bju.15830

**Published:** 2022-07-08

**Authors:** Ajay Aggarwal, Lu Han, Alison Tree, Daniel Lewis, Tom Roques, Vijay Sangar, Jan van der Meulen

**Affiliations:** ^1^ Department of Health Services Research and Policy London School of Hygiene and Tropical Medicine London UK; ^2^ Department of Oncology Guy's and St Thomas' NHS Foundation Trust London UK; ^3^ Royal Marsden Hospital and The Institute for Cancer Research London UK; ^4^ Norfolk and Norwich NHS Foundation Trust Norwich UK; ^5^ The Christie NHS Trust and Manchester University NHS Foundation Trust Manchester UK; ^6^ Manchester University Manchester UK

**Keywords:** prostate cancer, centralization, travel time, patient choice, radical prostatectomy, radical radiotherapy, #PCSM, #ProstateCancer, #uroonc

## Abstract

**Objective:**

To assess the impact of centralization of prostate cancer surgery and radiotherapy services on the choice of prostate cancer treatment.

**Patients and Methods:**

This national population‐based study used linked cancer registry data and administrative hospital‐level data for all 16 621 patients who were diagnosed between 1 January 2017 and 31 December 2018 with intermediate‐risk prostate cancer and who underwent radical prostatectomy (RP) or radical radiation therapy (RT) in the English National Health Service (NHS). Travel times by car to treating centres were estimated using a geographic information system. We used logistic regression to assess the impact of the relative proximity of alternative treatment options on the type of treatment received, with adjustment for patient characteristics.

**Results:**

Of the 78 NHS hospitals that provide RT or RP for prostate cancer, 41% provide both, 36% provide RT and 23% provide RP. Compared to patients who had both treatment options available at their nearest centre where overall 57% of patients received RT and 43% RP, patients were less likely to receive RT if their nearest centre offered RP only and the extra travel time to a hospital providing RT was >15 min (52% of patients received RT and 48% RP%, odds ratio [OR] 0.70 (0.58–0.85); *P* < 0.001). Conversely, patients were more likely to receive RT if their nearest centre offered RT and the extra travel time to a hospital providing RP was >15 min (63% of patients received RT and 37% RP, OR 1.23 (1.08–1.40); *P* < 0.001). There was a negligible impact on the type of treatment received if centres providing alternative treatment options were ≤15‐min travel time from each other.

**Conclusion:**

The relative proximity of prostate cancer treatment options to a patient's residence is an independent predictor for the type of radical treatment received. Centralization policies for prostate cancer should not focus on one treatment modality but should consider all treatments to avoid a negative impact on treatment choice.

**Abbreviations**
HES: Hospital Episode Statistics
IMD: Index of Multiple Deprivation
IQR: interquartile range
LSOA: lower super output area
RCS: Royal College of Surgeons
RP: radical prostatectomy
RT: radiation therapy
RTDS: National Radiotherapy Data Set

## 1 Introduction

Cancer surgical services, particularly for prostate, pancreatic and oesophageal cancer, continue to be centralized to fewer high‐volume centres [1]. Whilst this may result in patients having to travel further for care [2], a move to greater centralization is based on evidence that centres performing a high volume of procedures have better patient outcomes following surgery [3]. In addition, treatments for cancer are becoming increasingly complex, often requiring one or more modalities of treatment (surgery, systemic anti‐cancer therapies, or radiotherapy), either in sequence or in combination. Therefore, it is important that these modalities are readily accessible for patients and embedded in predefined pathways of care.

Prostate cancer is almost unique as a tumour type because patients diagnosed with intermediate‐risk disease are potentially eligible for up to three curative treatments: radical prostatectomy (RP), external beam radiation therapy (RT) or brachytherapy [4]. Given that cure rates are similar for these modalities, patient preferences are key and treatment options should be appraised according to their disease and personal characteristics [5, 6].

The type of treatment received by patients diagnosed with prostate cancer may be influenced by hospital or health system factors rather than by patient‐level factors such as differences in disease severity, age or comorbidity [7]. Factors identified in the literature include the treatment services available at the diagnosing hospital [8], health insurance status [9], ethnicity [10], socioeconomic status [11], geographic region of residence [12], clinician bias and the availability of joint uro‐oncology consultations [13], and the distance patients have to travel [14].

When considering access to prostate cancer treatment, a potential barrier that has received little attention is that RT and RP, the mainstays of prostate cancer treatment, are often not co‐located at the patient's nearest treatment centre [15]. Patients who are eligible for either treatment option therefore need to consider the relative proximity to each of these options when deciding about which treatment they wish to receive.

In this national population‐based study in the English NHS, we first assessed the availability of RT and RP in the nearest prostate cancer treatment centre for patients with intermediate‐risk prostate cancer. Second, we investigated the impact of the relative proximity of these alternative treatment options on the type of prostate cancer treatment received by considering the extra travel burden when either RT or RP was not available in the nearest centre.

## 2 Patients and Methods

### 2.1 Data Sources and Patient Population

All 91 207 patients who were newly diagnosed with prostate cancer between 1 January 2017 and 31 December 2018, according to the English Cancer Registry using the International Classification of Diseases‐10 diagnosis code C61 were eligible for inclusion. Prostate cancer risk was based on TNM stage [16], Gleason score, and PSA level, according to a modified D'Amico risk stratification algorithm developed previously by the National Prostate Cancer Audit [17]. We excluded 5213 patients because they had low‐risk disease, 34 055 because they had locally advanced disease, and 12 463 because they had metastatic disease.

The English Cancer Registry data were linked at the patient level with Hospital Episode Statistics (HES), an administrative database of all hospital episodes in the English NHS [18] and the National Radiotherapy Data Set (RTDS). The OPCS Classification of Interventions and Procedures (OPCS‐4) code ‘M61’ in the HES database was used to identify patients who underwent RP and the date of their operation. The RTDS data item ‘treatment modality’ was used to select patients who underwent external beam RT and the date of their treatment. A patient was considered to have undergone radical prostate cancer therapy if he was identified as having received RP or RT within 12 months of his diagnosis date.

**Fig. 1:**
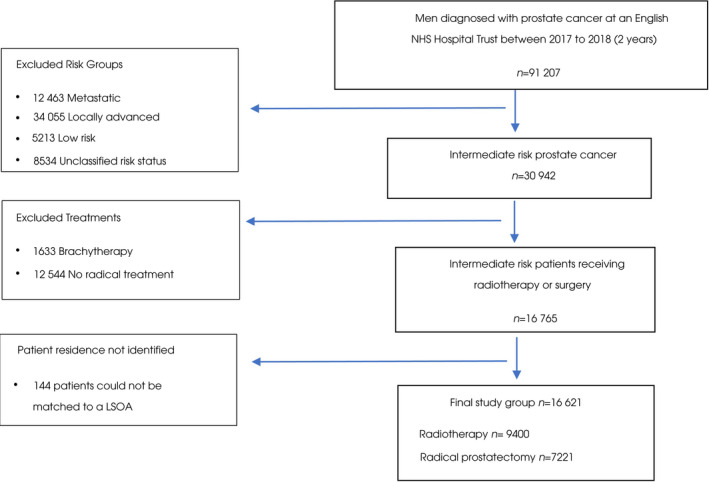
Consort diagram of patient selection. LSOA, lower super output area.

Of the 30 942 patients identified with intermediate‐risk disease, 1633 were excluded because they received brachytherapy and 12 544 were excluded because they did not have a recorded treatment, representing patients on active surveillance or watchful waiting **(**Fig. 1 **)**. A total population of 16 621 patients were eligible for analysis.

### 2.2 Baseline Characteristics

Patients were categorized into the ethnic groups Asian, Black, Mixed, White, or Other, as defined in the 2001 Census in England and Wales [19]. The Royal College of Surgeons (RCS) Charlson score was used to identify any comorbid conditions captured in the HES record [20]. Socioeconomic deprivation status was categorized into groups according to the national quintiles of the ranking of the English 2010 Index of Multiple Deprivation (IMD) rankings of the 32 844 lower super output areas (LSOAs). LSOAs contain typically 1500 residents or 650 households [21]. Least deprived and most deprived patients were defined as the lowest two and highest three quintiles of IMD, respectively.

### 2.3 Travel Times

In the analysis we used travel time to capture the distance between a patient's residence and each prostate cancer treatment centre in the English NHS. The population‐weighted centroids of the LSOAs of the patients' residence and the full postal codes for the hospitals providing RT or RP were used to calculate travel times according to the fastest route by car (using Ordnance Survey MasterMap Highways Network). For each patient, the difference in travel time between their nearest surgical and radiotherapy centre was calculated.

In the analysis we considered three groups: Group 1: patients who had both RT and RP available at their nearest prostate cancer treatment centre; Group 2: patients who only had RT available at their nearest treatment centre; and Group 3: patients who only had RP available at their nearest treatment centre. The travel time difference for Group 1 was 0 min and for Groups 2 and 3 represented the extra travel time from the patient's nearest prostate cancer treatment centre to the centre offering the alternative treatment modality.

Travel time categories for the analysis were based on the distribution of the difference in travel times between a patient's residence and their nearest RP and RT centre. It was also important to select categories that were relevant to inform clinical practice and policy. Of the patients in Group 2, who only had RT available at their nearest centre (*n* = 5160), 28.4% had an RP centre an additional 15 min further away and 12.8% had an RP provider an additional 30 min away, with a maximum travel time difference of 90 min. Of the patients in Group 3, who only had RP available at their nearest centre (*n* = 3644), 16.2% had an RT centre an additional 15 min away and 2.3% had an RT centre an additional 30 min away, with a maximum travel time difference of 40 min.

To ensure there was sufficient statistical power between the comparison groups a 15‐min cut‐off was chosen. In addition, this represented an extra travel burden that was felt likely to be significant to patients when making decisions about what treatment to have and in what centre compared to lower travel time categories.

Patients were therefore allocated to one of five categories depending on what services were available at their nearest prostate cancer treatment centre and the extra travel time to centres offering the alternative treatment option. The categories are defined below:

1. Both RP and RT available at their nearest centre (no travel time difference);
2. Only RT available at the nearest centre and the extra travel time to a hospital providing RP is more than 15 min;
3. Only RT available at the nearest centre and the extra travel time to a hospital providing RP is less than 15 min;
4. Only RP available at the nearest centre and extra travel time to a hospital providing RT is less than 15 min;
5. Only RP available at the nearest centre and the extra travel time to a hospital providing RT is more than 15 min.

### 2.4 Statistical Analysis

Data were complete for age, comorbidity, and socioeconomic status. In our final population, 6.7% of patients had missing ethnicity data. Missing values for ethnicity were not imputed as there could be a systematic rather than a random mechanism underlying missingness [22]. Patients with missing ethnicity data were therefore assigned to a separate category.

Multivariable logistic regression was used to estimate the association between the actual treatment (RP or RT) and the relative proximity of the two treatment options to a patient's residence according to the five travel time categories outlined above. The baseline comparison group was patients who had both treatment options (RP and RT) available at their nearest centre. The regression model was used to test whether the differences in the odds of receiving RT as the travel time difference increased or decreased was statistically significant. *P* values smaller than 0.05 were considered statistically significant. An odds ratio (OR) larger than 1 indicated that the odds of receiving RT was larger and an OR smaller than 1 indicated that the odds of receiving RT was smaller than when RT and RP were both offered in the nearest hospital.

We adjusted for the following patient characteristics in the regression model: age, comorbidity, socioeconomic status and ethnicity. These variables were included as categorical variables. A sensitivity analysis was also undertaken restricting the study population to patients aged 75 years and under, given that very few operations are undertaken in the age category of over 75 years.

As an additional analysis, we used interaction terms to assess to what extent the observed associations between the relative proximity of the two treatment options and the treatment they actually received varied according to four patient characteristics: age (patients older than 70 vs patients aged 70 or younger); comorbidity (patients with one or more comorbidity as defined by the Charlson comorbidity index vs patients with no comorbidity); socioeconomic status (patients from more deprived backgrounds (IMD 3–5) vs patients from less deprived backgrounds (IMD 1–2); ethnicity (patients from ethnic minority groups vs White patients). Wald tests were performed to test the statistical significance of the interaction terms. stata version 14 was used to undertake the statistical analyses (StataCorp, College Station, TX, USA).

Ethics approval for use of secondary anonymized patient‐level datasets for these analyses was received from the NHS Research Ethics Committee on 1 June 2020, reference: 20/WA/0161.

## 3 Results

Of the 16 621 patients with intermediate‐risk prostate cancer, 7221 (43.4%) received RP and 9400 (56.6%) received RT (Table 1). The median (interquartile range [IQR]) age for patients who received RP was 63 (57–68) years, and 71 (IQR 65–75) years for those who received RT. Approximately 15% of the study population had one or more comorbidities, and 8% were from an ethnic minority background. Patients receiving RT were older and had a greater number of comorbidities compared to patients receiving RP.

**Table 1:** Distribution of patient characteristics and proximity of radical treatment services.

	Total	%	Received radical treatment		*P*
	*n* = 16 621		RP (*n* = 7221)	RT (*n* = 9400)	χ^2^
**Age**					
<60 years	3439	20.7	2543 (35.2)	896 (9.5)	<0.001
60–64 years	2751	16.6	1619 (22.4)	1132 (12.0)	
65–69 years	4148	25	1989 (27.5)	2159 (23.0)	
70–74 years	3803	22.9	985 (13.6)	2818 (30.0)	
≥75 years	2480	14.9	85 (1.2)	2395 (25.5)	
**Comorbidities, using RCS Charlson score**					
0	13 565	81.2	6311 (87.4)	7254 (77.1)	<0.001
1	2338	14.07	774 (10.7)	1564 (16.6)	
2+	718	4.3	136 (1.9)	582 (6.2)	
**Deprivation status: national quintiles**					
1 (least deprived)	4326	26	1842 (25.5)	2484 (26.4)	0.200
2	4045	24.3	1739 (24.1)	2306 (24.5)	
3	3425	20.6	1487 (20.6)	1938 (20.6)	
4	2827	17	1283 (17.8)	1544 (16.4)	
5 (most deprived)	1998	12	870 (12.1)	1128 (12)	
**Ethnicity**					
Asian	318	1.9	142 (2.0)	176 (1.9)	<0.001
Black	789	4.8	485 (6.7)	304 (3.2)	
Mixed	93	0.6	59 (0.9)	34 (0.4)	
White	14 107	84.9	5924 (82.04)	8183 (87.1)	
Other±	197	1.2	103 (1.4)	94 (1.0)	
Missing	1117	6.7	508 (7.0)	609 (6.5)	
**Ethnicity**					
White	14 107	84.9	5924 (82.0)	8183 (87.1)	<0.001
Ethnic minority group	1397	8.4	1194 (10.9)	1123 (6.5)	
Missing	1117	6.7	508 (7.0)	609 (6.5)	
**Treatment options available at nearest centre**					
Both RT and RP	7817	47	3337 (46.2)	4480 (47.7)	<0.001
Only RT available and nearest centre offering RP >15 min extra travel time away	1466	8.8	546 (7.6)	920 (9.8)	
Only RT available and nearest centre offering RP <15 min extra travel time away	3694	22.2	1650 (22.9)	2044 (21.7)	
Only RP available and nearest centre offering RT <15 min extra travel time away	3052	18.4	1404 (19.4)	1648 (17.5)	
Only RP available and nearest centre offering RT >15 min extra travel time away	592	3.6	284 (3.9)	308 (3.3)	

Of the 78 NHS hospitals in England that provided RT or RP for prostate cancer during the study period, 32 (41%) provided both, 28 (36%) provided RT alone and 18 (23%) provided RP alone. Nationally, across all patients, the median (IQR) travel time by car to their nearest RP centre was 21.0 (13–33) min and to their nearest RT centre it was 20.7 (12–31) min.

For 47.0% of the patients (*n* = 7817), the nearest RT and RP service was in the same hospital. For 31.0% of the patients (*n* = 5160), an RT centre was closer than an RP centre and, conversely, for 22.0% (*n* = 3644), an RP centre was closer than an RT centre (Table 1).

Table 2 presents the actual proportions of patients receiving RT and RP according to both the relative proximity of these modalities to their residence and their sociodemographic characteristics. For patients who had both RT and RP at the nearest centre, we observed that 57% received RT and 43% received RP.

**Table 2:** Results of the univariate and multivariate analysis assessing the impact of patient characteristics and the relative proximity of radiotherapy and surgical services to a patient's residence on the choice of treatment modality.

	Proportion of men receiving RT	Proportion of men receiving RP	Unadjusted OR for receipt of RT	*P*	Adjusted OR for receipt of RT	*P*
**Treatment options available at nearest centre**	***n* (%)**	***n* (%)**				
Both RT and RP	4489 (57.3)	3337 (42.7)	1	<0.001	1	<0.001
Only RT available and nearest centre offering RP >15 min extra travel time away	920 (62.8)	545 (37.2)	**1.25 (1.11–1.40)**		**1.23 (1.08–1.40)**	
Only RT available and nearest centre offering RP <15 min extra travel time away	2044 (55.3)	1650 (44.7)	0.93 (0.85–1.01)		**0.90 (0.83–0.98)**	
Only RP available and nearest centre offering RT <15 min extra travel time away	1648 (54.0)	1404 (46.0)	**0.88 (0.80–0.95)**		0.93 (0.84–1.02)	
Only RP available and nearest centre offering RT >15 min extra travel time away	308 (52.0)	284 (48.0)	**0.8 (0.68–0.95)**		**0.70 (0.58–0.85)**	
**Age**						
<60 years	896 (26.1)	2543 (73.9)	1	<0.001	1	<0.001
60–64 years	1132 (41.1)	1619 (58.9)	**2.0 (1.79–2.22)**		**1.98 (1.78–2.21)**	
65–69 years	2159 (52.1)	1989 (47.9)	**3.1 (2.8–3.4)**		**3.03 (2.74–3.34)**	
70–74 years	2818 (74.1)	985 (25.9)	**8.2 (7.4–9.1)**		**8.05 (7.2–9.0)**	
≥75 years	2395 (96.6)	85 (3.4)	**79.3 (63.1–99.8)**		**78.9 (62.6–99.2)**	
**Comorbidities: RCS Charlson score**						
0	7254 (53.5)	6311 (46.5)	1	<0.001	1	<0.001
1	1564 (66.9)	774 (33.1)	**1.76 (1.61–1.94)**		**1.58 (1.42–1.75)**	
2+	582 (81.1)	136 (18.9)	**3.75 (3.10–4.53)**		**3.05 (2.48–3.75)**	
**Deprivation status: national quintiles**						
1 (least deprived)	2484 (57.4)	1842 (42.6)	1	0.3264	1	<0.001
2	2306 (57.0)	1739 (43.0)	0.96 (0.91–1.02)		1.04 (0.94–1.15)	
3	1938 (56.6)	1487 (43.4)	0.97 (0.89–1.07)		1.06 (095–1.17)	
4	1544 (54.6)	1283 (45.4)	0.90 (0.82–1.00)		1.08 (0.97–1.21)	
5 (most deprived)	1128 (56.5)	870 (43.5)	**0.96 (0.86–1.06)**		**1.36 (1.20–1.54)**	
**Ethnicity**						
White	8183 (58.0)	5924 (42.0)	1	<0.001	1	0.041
Ethnic minority group	1123 (43.5)	1194 (56.5)	**0.56 (0.50–0.62)**		**0.75 (0.66–0.86)**	
Missing	609 (54.5)	508 (45.5)	0.87 (0.77–1.01)		1.06 (0.92–1.22)	

OR, odds ratio; RCS, Royal College of Surgeons; RP, radical prostatectomy; RT, radiation therapy. Travel time – represents the difference in travel time between the patients nearest RP and RT centre. An OR >1 means that the chance of receiving RT is higher than when RT and RP are both offered in the nearest hospital. Statistically significant results in bold font.

Compared to having both RT and RP available at the nearest centre. We found that patients were less likely to receive RT (52% of patients received RT and 48% received RP) if the nearest centre offered RP only and the extra travel time to a hospital providing RT was more than 15 min (OR 0.70 [0.58–0.85]; *P* < 0.001). Conversely, patients were more likely to receive RT (63% of patients received RT and 37% received RP) if their nearest centre offered only RT and the extra travel time to a hospital providing RP was more than 15 min (OR 1.23 [1.08–1.40]; *P* < 0.001).

Where the extra travel time between the nearest RT and RP centre was less than 15 min, the differences in the proportions of men receiving RT or RP were small and largely insignificant compared to centres where both services were co‐located. If the nearest centre offered RT and the extra travel time to a hospital providing RP was less than 15 min, there was in fact a marginally lower chance of receiving RT (OR 0.90 [0.83–0.98]; *P* = 0.021). Similarly, there was no significant impact if the patients nearest hospital offered RP and the extra travel time to a hospital providing RT was less than 15 min (OR 0.93 [0.84–1.02]; *P* = 0.152).

The results in Table 2 further demonstrate that men were more likely to receive radical RT if they were from a more deprived socioeconomic background, were aged 70 years and above, or had one or more comorbidities (all *P* < 0.001). In addition, we found that men from minority ethnic backgrounds were less likely to receive radical RT (OR 0.75 [0.66–0.86]; *P* = 0.001). A sensitivity analysis restricting the population to men aged 75 years and under did not show any significant changes in the observed associations between travel time difference and the likelihood of receiving radical RT (Table S1).

**Fig. 2:**
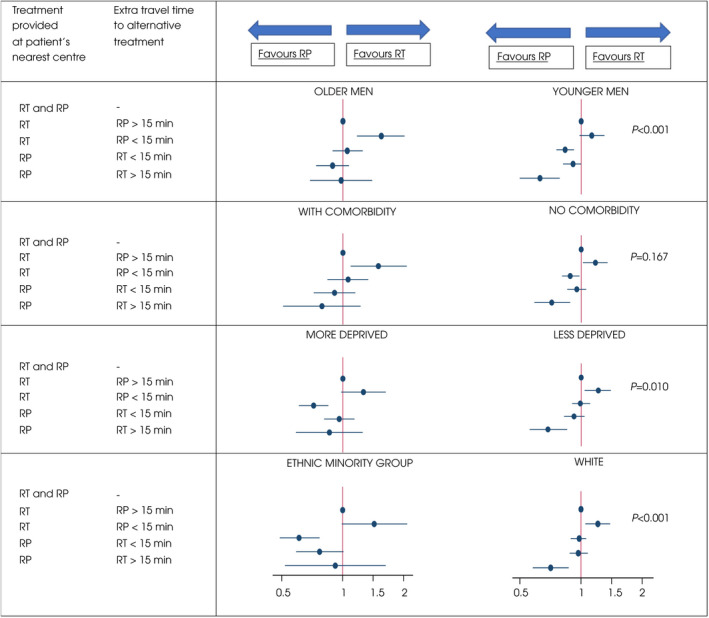
Plots of odds ratios (ORs) for the interactions between age, comorbidity, socioeconomic status and ethnicity on the association between the relative proximity of radiotherapy and surgical services to a patient's residence and the choice of treatment modality. The figure highlights equity differences between patient groups in the odds of receiving radiation therapy (RT) or radical prostatectomy (RP) according to the relative proximity of radiotherapy and surgical services to their residence. Interaction terms derived from a multivariable regression model were used to assess to what extent the observed associations between the relative proximity of the two treatment options and the treatment they actually received varied according to four patient characteristics: age (men older than 70 vs men aged 70 years or younger); comorbidity (men with one or more comorbidity as defined by the Charlson comorbidity index vs men with no comorbidity); socioeconomic status (men from more deprived backgrounds [IMD 3–5] vs men from less deprived backgrounds [IMD 1–2]); and ethnicity (men from ethnic minority groups vs White men). An OR >1 means that the chance of receiving RT is higher than when RT and RP are both offered at the nearest hospital (OR always 1 as it was the reference). The ORs are plotted with 95% CIs (horizontal lines). *P* values are presented, showing whether the differences observed between the plots within patient groups are statistically significant. For example, the association between age and relative proximity of treatment options on treatment choice varied when comparing the ORs for younger (age ≤ 70 years) and older men (age >70 years; *P* value <0.001).

We sought to understand whether the observed associations between travel time differences between the nearest RT and RP centre and the type of treatment patients received were influenced by patient characteristics. Travel time and patient characteristics were included in the regression model as interaction terms, adjusted for age, socioeconomic status, comorbidity and ethnicity (Fig. 2 and Table S2; see Methods for further explanation). These interactions were statistically significant apart from the interaction for comorbidity. Two patterns can be distinguished. First, across the four patient characteristics, there appeared to be a trend for patients who were younger, fitter (no comorbidity), from less deprived areas, and from a White ethnic background to be more likely to receive RP when the centre providing RT was more than 15 extra minutes away. Second, the opposite seemed to be the case for older patients and those who were less fit (with comorbidities), from more deprived areas, and from a minority ethnic background. These patients were consistently more likely to receive RT when the centre providing RP was more than an extra 15 min away.

## 4 Discussion

This is the first analysis that has sought to understand how the configuration of cancer services, specifically the relative proximity based on travel times to centres offering different prostate cancer treatment options, influences patients' choice of treatment. We found that over half of men requiring prostate cancer treatment in the NHS do not have both RT and RP services co‐located at their nearest treatment centre and that the relative proximity of these treatment options to a patient's residence is an independent predictor for the type of radical treatment received.

Patients were more likely to choose a treatment option available closest to them if the nearest centre offering the alternative treatment was over 15 min away. These findings therefore demonstrate how the configuration of specialist services in the English NHS can influence the type of cancer treatment patients receive, which could subsequently have a longer‐term impact on their functional and survival outcomes as well as their treatment experience [4, 23].

The observed associations were present even after adjusting for age, socioeconomic status, comorbidity and ethnicity. In addition, the associations between extra travel time and the treatment that patients received had little effect if the nearest centres offering the alternative treatments were within 15‐min travel time. This would suggest that patients may disregard the inconvenience of small additions in trip length but not perceived larger ones.

Patient characteristics also had an impact on the association between travel time and the choice of treatment, with younger men more likely to be deterred from receiving RT than older men if the nearest hospital provides RP and the alternative RT is at least 15 min further away. This may also reflect the decision making of patients and clinicians with respect to suitability for treatments. For instance, there is evidence that men aged 75 and over may experience worse functional outcomes following RP [24].

With respect to the wider literature, we identified two US studies that analysed the impact of travel distance on the likelihood of receiving RT or RP for prostate cancer. The first study demonstrated using a nationwide dataset, that as the distance from the patient residence to the nearest RT facility increased, the proportion of patients receiving RT decreased (53.3% *≤*5 miles vs 33.8% >15 miles; *P* < 0.001 [14]), which is in line with our results. The implications with regard to receipt of RP were not explicitly explored in this study. Another study, using a state‐level dataset, found no association between travel time and utilization of RT or RP [25].

Whilst these findings are important, our study moves beyond a more traditional assessment of access, which tends to focus on utilization rates of a single treatment modality rather than the inter‐dependency between treatment options and their relative proximity. Our findings are particularly pertinent for prostate cancer, where centralization of RP services in the English NHS has been occurring at pace (the number of RP centres has decreased from 65 to 51 in the last 10 years), with little or no change in the number of RT centres given the patient volumes and the necessity for these centres to deliver treatment across all tumour types [26].

Currently, only 40% of all NHS hospitals providing prostate cancer treatment in England are “comprehensive cancer centres” whereby RP and RT services for prostate cancer are available at the same site. Whilst the creation of a comprehensive centre presents an ideal solution, the reality is that not all services can be co‐located. However, our findings should inform future centralization initiatives because they show that there are potentially thresholds in the travel time trade‐off effects for cancer patients who are eligible for more than one curative treatment modality.

Furthermore, our finding that older and less fit patients were less likely to have RP if the centre providing prostate surgery was more than an extra 15‐min travel time away demonstrates that we need to ensure that centralization does not disproportionately affect access to specific demographic groups. One approach to avoid this is to undertake an empirical assessment of the potential impact of policy initiatives aiming to centralize or reconfigure services to ensure that the inherent extra travel burden does not disproportionately affect vulnerable groups, for whom barriers to access to cancer treatment are well documented [8]. Methods for this type of policy assessment have previously been presented [2]. In addition, there should be a clear objective as to what the centralization seeks to achieve as part of the planning approach to ensure these trade‐offs are described transparently before decisions are made (e.g., better patient experience, improvement in quality, greater efficiency in use of workforce and capital expenditure on equipment).

Whilst our study does highlight that the configuration of specialist prostate cancer treatment services impacts on the treatment that men with prostate cancer receive, overcoming access barriers by changing the structure or design of the service is a complex and expensive approach. Therefore, short‐term options to overcome these barriers may include the greater use of joint uro‐oncology clinics [27], patient pathway navigators, or electronic decision aids [28]. For those without access to a car, subsidized or free hospital transport is also an important consideration. In addition, recent evidence has demonstrated that reducing the number of visits required for prostate cancer radiotherapy, for example, through the introduction of shorter hypofractionated treatments, can improve equitable access to treatment and reduce the cost implications of travel [29, 30].

Of note, we found that patients from ethnic minority backgrounds were more likely to receive RP than RT. This is in part likely to relate to the younger age of these men compared to White men and explains why the association was attenuated in the fully adjusted regression model when including other patient characteristics. However, further qualitative work is needed to understand potential factors underpinning differences in treatment preference in patients from different ethnic minority backgrounds.

The strengths of this population‐based study in the English NHS include the large number of patients included, which underlines the representativeness of our results for a state‐funded health service, and the high level of accuracy and completeness of most of the routinely collected data items.

Our analytical approach used average drive times, which is the standard measure of travel distance for this kind of analyses and is considered superior to using straight‐line distances. However, we acknowledge that drive times are variable depending on the time of day, which may affect patients' decision making. We also undertook a sensitivity analysis (not presented) using public transport times. Although travel times by public transport are typically longer than drive times, the observed associations were not different. In this analysis we used a threshold travel time difference between treatment options of 15 min. This was based on the distribution of travel time differences nationally and directly relates to the configuration of prostate cancer treatment services in England. These thresholds would potentially need to be adapted for analyses in different country settings depending on the configuration of services.

We did not include brachytherapy treatments given the small proportion of patients receiving this treatment relative to RP and RT in the English NHS. In addition, previous work has highlighted that the highly centralized nature of brachytherapy services in the NHS has resulted in access deficits [8, 23].

In terms of limitations, there will be factors not available in our dataset which may have an impact the type of treatment that was received, including the use of public or hospital transport and perceptions of hospital quality [31, 32]. However, we have sought to minimize the impact of residual confounding by both adjusting for important patient characteristics that may be associated with differences in how patients access care or their suitability for particular treatment (i.e., age, comorbidity, socioeconomic status and ethnicity) and including only men diagnosed with intermediate‐risk prostate cancer (thereby minimizing the impact of cancer risk on our findings) [10].

In conclusion, we demonstrated that the configuration of prostate cancer services and the relative proximity of centres providing RT and RP to the patients' place of residence was associated with the type of treatment that men with intermediate‐risk prostate cancer received. Patients were more likely to choose a treatment modality that was delivered closest to them. The results varied according to the patient's age, comorbidity, socioeconomic deprivation, and ethnic background, which demonstrates that centralization initiatives may affect equity in access to cancer services.

Policy initiatives focusing on centralizing prostate cancer services need to consider the potential impact of extra travel time on the type of cancer treatments that patients receive. Our results demonstrate that centralization policies for prostate cancer should not focus on one modality but should consider the full range of treatment modalities available to avoid a negative impact on treatment choice.

## Disclosure of Interests

Alison Tree reports research funding from Elekta, Varian and Accuray and Honoraria/travel assistance: Elekta, Accuray, Janssen. All other authors have no disclosures of interest.

## Supporting information


**Table S1** Results of the univariate and multivariate analysis for patients aged 75 and under, assessing the impact of patient characteristics and the relative proximity of radiotherapy and surgical services to a patient’s residence on the choice of treatment modality.
**Table S2** Table of the odds ratios for the interactions between age; comorbidity; ethnicity; and socioeconomic status on the association between the relative proximity of radiotherapy and surgical services to a patients residence on the choice of treatment modality.
