# Supplementary material for: Impact of centralization of prostate cancer services on the choice of radical treatment
Source: BJU Int. 2022 Jul 8;131(1):53–62. doi: 10.1111/bju.15830 (PMC10084068; doi:10.1111/bju.15830)
Supplement: Supplementary file 1 — Table S1 Results of the univariate and multivariate analysis for patients aged 75 and under, assessing the impact of patient characteristics and the relative proximity of radiotherapy and surgical services to a patient’s residence on the choice of treatment modality. Table S2 Table of the odds ratios for the interactions between age; comorbidity; ethnicity; and socioeconomic status on the association between the relative proximity of radiotherapy and surgical services to a patients residence on the choice of treatment modality. [file BJU-131-53-s001.pdf]

Appendix Table 1 - Results of the univariate and multivariate analysis for patients aged 75 and under, assessing the impact of patient characteristics and the relative proximity of radiotherapy and surgical services to a patient's residence on the choice of treatment modality.

	**Proportion of men treated with radiotherapy**	**Proportion of men treated with radical prostatectomy**	**Unadjusted odd ratios for receipt of radiotherapy**		**Adjusted odds ratio for receipt of radiotherapy**	
			**95% CI**	***P* values**		
**Treatment options available at nearest centre**				<0.001		<0.001
Both RT and RP	57.3%	42.7%	reference		reference	1
Only RT available and nearest centre offering RP >15 mins extra travel time away	62.8%	37.2%	**1.28 (1.14-1.44)**		**1.24 (1.09-1.41)**	
Only RT available and nearest centre offering RP <15 mins extra travel time away	55.3%	44.7%	**0.89 (0.82-0.97)**		**0.89 (0.81-0.97)**	
Only RP available and nearest centre offering RT < 15 mins extra travel time away	54%	46%	**0.90 (0.82-0.98)**		0.94 (0.85-1.03)	
Only RP available and nearest centre offering RT >15 mins extra travel time away	52%	48%	**0.78 (0.65-0.92)**		**0.70 (0.58-0.85)**	
**Age (Years)**				<0.001		<0.001
<60	26.1%	73.9%	reference		reference1	
60-64	41.1%	58.9%	**1.98 (1.78-2.21)**		**1.98 (1.78-2.20)**	
65-69	52.1	47.9%	**3.08 (2.79-3.40)**		**3.02 (2.74-3.34)**	
70-74	74.1	25.9%	**9.48 (8.54-10.50)**		**9.35 (8.41-10.40)**	
**Comorbidities (using RCS Charlson Score)**				<0.001		<0.001
0	53.5%	46.5%	1		1	
1	66.9%	33.1%	**1.76 (1.61-1.94)**		**1.59 (1.44-1.77)**	
2+	81.1%	18.9%	**3.75 (3.10-4.53)**		**3.15 (2.56-3.88)**	
**Deprivation status (national Quintiles)**				0.4931		<0.001
1 (least deprived)	57.4%	42.6%	1		1	
2	57.0%	43.0%	1.01 (0.92-1.10)		1.03 (0.94-1.14)	
3	56.6%	43.4%	1.00 (0.91-1.10)		1.05 (0.95-1.17)	
4	54.6%	45.4%	0.94 (0.85-1.04)		1.05 (0.94-1.18)	
5 (most deprived)	56.5%	43.5%	1.04 (0.93-1.16)		**1.19 (1.20-1.52)**	
**Ethnicity**				<0.001		0.001
White	58.0%	42%	1		1	
Ethnic minority group	43.5%	56.5%	**0.56 (0.50-0.62)**		**0.75 (0.66-0.86)**	
Missing	54.5%	45.5%	0.88 (0.77-1.01)		1.08 (0.94-1.24)	

Notes:

- RT – Radiotherapy; RP -Radical prostatectomy
- Travel time – represents the difference in travel time between the patients nearest RP and RT centre.
- An Odds Ratio (OR) > 1 means that the chance of receiving RT is higher than when RT and RP are both offered in the nearest hospital.
- Statistically significant results in bold font

Appendix Table 2. Table of the odds ratios for the interactions between age; comorbidity; ethnicity; and socioeconomic status on the association between the relative proximity of radiotherapy and surgical services to a patients residence on the choice of treatment modality.

	**Odds Ratio**		**Odds Ratio**		LR Test
	**n=16424**		**95% CI**		***P* values**
**Treatment options available at nearest centre**	**Older men**		**Younger men**		**<0.001**
Both RT and RP	1		1		
Only RT available and nearest centre offering RP >15 mins extra travel time away	1.54	1.17-2.00	1.13	0.98-1.31	
Only RT available and nearest centre offering RP <15 mins extra travel time away	1.05	0.89-1.25	0.83	0.75-0.92	
Only RP available and nearest centre offering RT < 15 mins extra travel time away	0.89	0.74-1.07	0.91	0.81-1.00	
Only RP available and nearest centre offering RT >15 mins extra travel time away	0.98	0.69-1.39	0.62	0.49-0.78	
**Treatment options available at nearest centre**	**With comorbidity**		**No Comorbidity**		**0.167**
Both RT and RP	1		1		
Only RT available and nearest centre offering RP >15 mins extra travel time away	1.49	1.09-2.05	1.18	1.02-1.36	
Only RT available and nearest centre offering RP <15 mins extra travel time away	1.06	0.84-1.33	0.88	0.80-0.98	
Only RP available and nearest centre offering RT < 15 mins extra travel time away	0.91	0.72-1.15	0.95	0.85-1.06	
Only RP available and nearest centre offering RT >15 mins extra travel time away	0.79	0.51-1.22	0.71	0.58-0.88	
**Treatment options available at nearest centre**	**Most socioeconomically deprived**	**95% CI**	**Least socioeconomically deprived**	**95% CI**	**0.01**
Both RT and RP	1		1		
Only RT available and nearest centre offering RP >15 mins extra travel time away	1.26	0.98-1.62	1.22	1.04-1.41	
Only RT available and nearest centre offering RP	0.72	0.61-0.85	0.99	0.90-1.11	

<15 mins extra travel time away					
Only RP available and nearest centre offering RT < 15 mins extra travel time away	0.96	0.81-1.14	0.92	0.82-1.04	
Only RP available and nearest centre offering RT >15 mins extra travel time away	0.86	0.59-1.25	0.68	0.55-0.85	
**Treatment options available at nearest centre**	**Ethnic minority group**		**White**		**<0.001**
Both RT and RP	1		1		
Only RT available and nearest centre offering RP >15 mins extra travel time away	1.43	0.99-2.08	1.21	1.05-1.39	
Only RT available and nearest centre offering RP <15 mins extra travel time away	0.61	0.49-0.77	0.98	0.89-1.06	
Only RP available and nearest centre offering RT < 15 mins extra travel time away	0.77	0.59-1.01	0.97	0.88-1.08	
Only RP available and nearest centre offering RT >15 mins extra travel time away	0.92	0.52-1.63	0.71	0.58-0.87	

Notes:

RT – Radiotherapy; RP - Radical prostatectomy

Younger men defined as age <=70. Older Men age >70.

Most deprived – men living in areas of highest social deprivation (IMD score 3-5). Least deprived – men living in areas of lowest social deprivation (IMD 1-2).

Travel time – represents the difference in travel time between the patients nearest RP and RT centre.

An Odds Ratio (OR) > 1 means that the chance of receiving RT is higher than when RT and RP are both offered at the nearest hospital.
